# Ski Mountaineering: Perspectives on a Novel Sport to Be Introduced at the 2026 Winter Olympic Games

**DOI:** 10.3389/fphys.2021.737249

**Published:** 2021-10-21

**Authors:** Lorenzo Bortolan, Aldo Savoldelli, Barbara Pellegrini, Roberto Modena, Massimiliano Sacchi, Hans-Christer Holmberg, Matej Supej

**Affiliations:** ^1^Department of Neurosciences, Biomedicine and Movement Sciences, University of Verona, Verona, Italy; ^2^CeRiSM, Sport Mountain and Health Research Centre, University of Verona, Rovereto, Italy; ^3^Oberalp S.p.A., Bolzano, Italy; ^4^Department of Health Sciences, Luleå University of Technology, Luleå, Sweden; ^5^Faculty of Sport, University of Ljubljana, Ljubljana, Slovenia

**Keywords:** winter sport, sport performance, endurance, Olympic sport, athletes, training and testing

## Abstract

Ski mountaineering is a rapidly growing winter sport that involves alternately climbing and descending slopes and various racing formats that differ in length and total vertical gain, as well as their distribution of downhill and uphill sections. In recent years, both participation in and media coverage of this sport have increased dramatically, contributing, at least in part, to its inclusion in the 2026 Winter Olympics in Milano-Cortina. Here, our aim has been to briefly describe the major characteristics of ski mountaineering, its physiological and biomechanical demands, equipment, and training/testing, as well as to provide some future perspectives. Despite its popularity, research on this discipline is scarce, but some general characteristics are already emerging. Pronounced aerobic capacity is an important requirement for success, as demonstrated by positive correlations between racing time and maximal oxygen uptake and oxygen uptake at the second ventilatory threshold. Moreover, due to the considerable mechanical work against gravity on demanding uphill terrain, the combined weight of the athlete and equipment is inversely correlated with performance, prompting the development of both lighter and better equipment in recent decades. In ski mountaineering, velocity uphill is achieved primarily by more frequent (rather than longer) strides due primarily to high resistive forces. The use of wearable technologies, designed specifically for analysis in the field (including at elevated altitudes and cold temperatures) and more extensive collaboration between researchers, industrial actors, and coaches/athletes, could further improve the development of this sport.

## Introduction

Ski mountaineering, a winter sport that involves alternately climbing and descending slopes, has become increasingly popular among both recreational and competitive athletes, presumably because of the demanding endurance exercise associated with the climbs and the excitement of descents on largely unprepared slopes (“off-piste” skiing), as well as the spectacular closeness to nature.

As with other skiing disciplines, the early development of ski mountaineering is indicated by prehistoric images depicting individuals traveling across the snow on wooden boards, as well as by Middle Age paintings showing how animal skins were placed on skis to facilitate climbing uphill (Street, 1992).

The first ski mountaineering competition was held in Italy in 1933 and, during the final decades of the twentieth century, the number of ski mountaineering races involving different levels of performance (recreational to elite) rose markedly. At present, the International Ski Mountaineering Federation (ISMF), to which 38 nations belong, regulates and promotes this sport worldwide, organizing World Cup events and World Championships. In July 2021, the International Olympic Committee decided to allow ski mountaineering as an event at the coming 2026 Winter Olympics in Milano-Cortina.

As a result, interest in ski mountaineering will grow, more will participate, and the Olympic organizations in different countries, as well as the sporting industry, will invest more in this area. These considerations motivated us to examine the major characteristics of competitive ski mountaineering here, as well as to explore the extensive potential for future scientific and technological development.

## Official Competitions

Regulated by the ISMF, there are five different types of official ski mountaineering competitions that differ primarily with respect to total vertical gain, total distance, and the pattern of downhill and uphill sections. These competitions are either individual (Sprint, Vertical, and “Individual”) or team races (“Team” and Relay), with “Individual” and “Team” being the major forms (Table 1). Among these, the Olympic committee decided to include only five: two sprint events, two individual events (one each for men and women), and one mixed-gender relay.

**TABLE 1:** General features of ISMF races and publications that deal with their characteristics.

**Form of competition**	**Categories**		**Total ascent**	**Duration**	**Competitors**	**Characteristics**	(Lasshofer et al., 2021)	(Portillo and Rodríguez, 2020)	(Gasser, 2019a)	(Gasser, 2019b)	(Gaston et al., 2019)	(Fornasiero et al., 2018)	(Praz et al., 2015)	(Praz et al., 2014)	(Duc et al., 2011)	(Schenk et al., 2011)	(Diaz et al., 2010)	(Durand et al., 2005)
Sprint	S	M/W	< 80 m	180–210 s	Individual	● A varied, short course with ascent, descent, and a section walking with skis attached to the backpack. ● Qualifying phases, quarterfinals, semi-finals, and final. ● Quarterfinal, semi-finals, and final heats involving six skiers each.		X										
	U23	M/W																
	U20	M/W																
	U18	M/W																

Vertical	S	M/W	500–700 m	20–30 min	Individual	● A single ascent on skis. ● No section walking. ● Can take place off-piste, but only along a sheltered track (min. 2 m width) ● The average gradient should be at least 15%.	X	X			X	X						
	U23	M/W																
	U20	M																
	U20	W	400–500 m															
	U18	M/W																

Individual	S	M	1600–1900 m	1.5–2 h	Individual	● Minimum of three ascents and descents. ● The length of the longest ascent may not exceed 50% of the total ascent. ● Out of the total difference in height (positive + negative): ● at least 85% must be covered on skis ● at most 5% can be covered on foot (footpaths, forest tracks, etc.) ● at most 10% should be technical sections covered carrying skis strapped to the backpack (ridges, couloirs, etc.)		X			X				X			X
	U23	M																
	S	W	1300–1600 m															
	U23	W																
	U20	M																
	U20	W	900–1200 m															
	U18	M/W	800–1000 m															
Team	S	M	> 2100 m	< 3 h (first team)	2				X	X			X	X		X	X	
	S	W	> 1800 m															

Relay	S	M	150–180 m	< 15 min	4	● Two separate ascents and descents covered by each member of the team, during the second ascent in part on foot with the skis strapped to the backpack.												
	S	W			3													
	Youth	M/W			3													

*S: senior; U23: under 23 years of age; U20: under 20 years of age; U18: under 18 years of age; Youth: young people; M: men; W: women.*

This sport requires both pronounced physiological capacity when skiing uphill (Duc et al., 2011; Schenk et al., 2011) and remarkable downhill skiing technique.

When traveling uphill, the skier must attach special skins under the skis, designed to prevent sliding backward. Prior to skiing downhill, the skins must be removed to reduce friction with the snow and thus increase speed, and both bindings and boots must be locked, all within well-defined transition areas. With the exceptions of Vertical and Relay races, all of these forms of competition include certain sections that must be covered walking with the skis tied to the backpack.

In contrast to other winter sports, the ISMF guidelines do not set limits on the altitude at which ski mountaineering competitions are arranged and many take place up to more than 3000 m or even, sometimes, 4000 m above sea level.

## Physiological Determinants

Research on the physiological determinants of performance has revealed this sport to be highly strenuous and energy-demanding (Duc et al., 2011; Schenk et al., 2011; Praz et al., 2014; Gaston et al., 2019). Duc et al. (2011) reported that during an “Individual” race, approximately half of the total racing time was spent between VT1 and VT2 (the first and second ventilatory thresholds, respectively), and another 40% above VT2, with an average heart rate (aHR%) of 93% of HRmax. During a team race (which is longer in duration), more than 70% of the total racing time was spent between VT1 and VT2 and 20% above VT2, with an aHR% of 87% (Schenk et al., 2011).

In their analysis of two races each lasting for more than 5 h, Praz and co-workers (2014) found that only a small fraction of the total time was spent above VT2 (17 ± 23% and 13 ± 20%), with an aHR% above 80% (81 ± 2% and 82 ± 4%); whereas during a simulated vertical race, Lasshofer et al. (2021) observed a higher aHR% (92 ± 2%). This negative relationship between intensity (as reflected in the percentage of the time spent above VT2 and aHR%) and race duration was further confirmed by Gaston et al. (2019), who observed aHR% of 93 ± 3% and 91 ± 2% during a “vertical” and “Individual” race, respectively. Furthermore, even during the downhill skiing, the aHR% remains high, around 85% (Duc et al., 2011), most likely due to pronounced activation of the leg muscles, as well as the psycho-emotional and physical stress associated with choosing the optimal trajectory on the challenging course with varying snow conditions.

Extensive aerobic capacity (VO_2__*max*_) appears to be a major determinant of success in ski mountaineering (Schenk et al., 2011; Fornasiero et al., 2018). During a simulated competition, the aHR% and mean VO_2_/VO_2__*max*_ of elite and sub-elite male skiers did not differ, whereas both the VO_2__*m*__*ax*_ and VO_2_ at VT2 of the former were significantly greater (elite: 71.2 ± 6.8 and 62.6 ± 5.4 ml⋅min^–1^⋅kg^–1^ versus sub-elite: 62.5 ± 4.7 and 55.3 ± 4.6 ml⋅min^–1^⋅kg^–1^, respectively) (Lasshofer et al., 2021). Indeed, Duc et al. (2011) observed a negative correlation between racing time and VO_2__*max*_, VO_2_ at VT1, and VO_2_ at VT2 relative to body mass. Moreover, Fornasiero et al. (2018) reported that most of the difference in the uphill performance of ski mountaineers could be explained by differences in their oxygen consumption relative to body mass at VT2.

With respect to general physiological requirements, ski mountaineering resembles other endurance disciplines such as cross-country skiing, (Duc et al., 2011). At the same time, it is important to remember that the length and distribution of phases of recovery during ski mountaineering differ from those in other skiing disciplines, with longer ascents and descents than, for example, in cross-country skiing (Sandbakk and Holmberg, 2017).

Moreover, the other ski endurance events at the Olympics are limited to a maximal altitude of 1800 m above sea level, whereas ski mountaineering is frequently performed at higher elevations, which involves more extensive hypoxia. It is well known that an athlete’s VO_2__*max*_ is reduced by approximately 6% for every 1000 m gain in elevation, an effect that is actually more pronounced for well-trained athletes (MacInnis et al., 2015) and persists even in competitive ski mountaineers acclimatized to elevated altitude (Faiss et al., 2014). This should be taken into account when using HR to assess the energy demands of ski mountaineering at different altitudes by applying the VO_2_/HR relationship (Richalet, 2012; Praz et al., 2014).

Furthermore, although the generally lower temperatures at elevated altitudes attenuate sweating, the loss of water connected with breathing is enhanced because the air is drier (Butterfield et al., 1992; Mawson et al., 2000). This can require special care to maintain adequate hydration. An additional challenge is the pronounced energy deficit that may arise because of the high demands placed on ski mountaineers, especially during competitions of longer duration (Praz et al., 2014).

Moreover, the influence of body composition on the uphill performance of ski mountaineers is demonstrated clearly by the finding that race time is correlated negatively to fat mass and body fat percentage (Schenk et al., 2011; Fornasiero et al., 2018). In cross-country skiing, the more extensive skiing at high speed on more well-prepared snow and less hilly terrain requires high strength and muscle mass and considerable involvement of the upper body (Pellegrini et al., 2018). Consequently, heavier cross-country skiers appear to have an advantage on all types of terrain except steep uphill and total body mass is not related to overall racing performance (Bergh and Forsberg, 1992), which is especially true for sprint specialists, who are more massive than long-distance cross-country skiers (Losnegard and Hallén, 2014).

## Biomechanical Aspects

In general, both stride frequency and length are major determinants of speed in bipedal locomotion. Skiing uphill during ski mountaineering is similar to diagonal stride in cross-country skiing, both involving the generation of propulsive force by upper- and lower-body muscles. In addition to helping generate force, poles help maintain balance and coordination (both uphill and downhill), as well as may reduce the cost of vertical locomotion and perceived exertion, as reported when using poles while trail running uphill (Giovanelli et al., 2019).

Higher speed on moderate uphill terrain is achieved by increasing both stride length and frequency, with a shortened propulsive phase, but no significant variation in its relative duration (Praz et al., 2016a, b). In comparison to cross-country skiing, much steeper inclines can be climbed by ski mountaineers as the result of the high friction between the skins (compared to grip wax) and the snow. On such steep inclines, both stride frequency and length are reduced to overcome gravity (i.e., increase potential energy). In contrast, cross-country skiing speed appears to be more dependent on stride length than frequency (Zoppirolli et al., 2020). Stride length in ski mountaineering is limited by the shorter distance covered by gliding due to the high friction produced by the skins and the less effective mechanical properties of the skis (geometry and stiffness), as well as the restriction of flexion-extension by the ski boots. Therefore, ski mountaineers should train on a variety of terrains under different snow conditions utilizing various stride frequencies and lengths (Lasshofer et al., 2021), as well as variations in technique.

Except in “vertical” events, all ski mountaineering courses contain at least one descent. Although the considerably shorter descent times exert less impact on the outcome of a race than ascent times, skiers who perform better uphill also appear to perform better downhill (Duc et al., 2011; Praz et al., 2014). Since the descents can involve a considerable variety of surface conditions, ranging from deep and fresh powder snow to moguls and hard ice, skiing these sections as rapidly as possible without falling requires a wide range of skills. During classic team competitions, when two or three skiers are connected by a climbing rope that forces them to coordinate their skiing, descents can be even more challenging.

These various factors require ski mountaineers to ski down slopes in a manner that is quite different from that utilized by competitors in the more well-known alpine disciplines (Hébert-Losier et al., 2014; Gilgien et al., 2018a). Since the low position (tuck/egg) used downhill in connection with these other disciplines (Barelle et al., 2004; Supej et al., 2013; Hébert-Losier et al., 2014) does not allow maintenance of good balance when turning on uneven ground and, moreover, is associated with more extensive muscle fatigue, this position is seldom, if ever, used by ski mountaineers. Moreover, unlike with alpine skiing (Supej et al., 2013), aerodynamic drag apparently exerts little influence on the downhill performance of ski mountaineers. Even though ski mountaineers sometimes reduce control, even on challenging descents, to lessen muscle fatigue (Gilgien et al., 2018b; Supej et al., 2018; Supej and Holmberg, 2019), the incidence of injuries in connection with this sport is relatively low compared to other winter sports (Palmer et al., 2021).

## Training

To date, only a single study has characterized the training of ski mountaineers who compete in national and international events, revealing an average weekly training of 16 ± 0.9 h and 50,814 ± 3,654 m of ascent annually (Durand et al., 2005). To obtain a better understanding of the training routines of senior and U23 athletes, we interviewed the coaches of the national ski mountaineering teams of Switzerland, Italy, and France. These coaches reported that, on average, their male athletes train 650–950 h annually, with 250–300,000 m of ascent, which is similar to the training volume of elite cross-country skiers (Sandbakk and Holmberg, 2017). During the preparatory and competition periods, the members of these national teams train 16–24 and 12–18 h weekly, respectively. For the female skiers, all of these volumes are 15–20% smaller. Training volume and program are influenced by the fact that some ski mountaineers, even among those belonging to a national team, have other employment.

Roughly 45% of the training by these ski mountaineers is on skis, the remainder usually involving cycling, running, hiking, and roller skiing (mainly during the summer/fall). Although most also train strength, relatively few designs this type of training on the basis of the races they specialize in. Coaches use training at elevated altitudes both to allow on-snow training during late summer and to prepare for competitions.

Ski mountaineers routinely assess their training with GNSS and HR monitors, and utilize HRV to assess their balance between stress and recovery, particularly during multi-race events (Portillo and Rodríguez, 2020).

## Testing

The extent and periodization of training are routinely based on fitness, as assessed by a variety of physiological tests (Jamnick et al., 2020). Many ski mountaineers utilized HR-based training zones to monitor exercise intensity. These zones are defined from the relationship between blood lactate concentration, HR, and oxygen consumption during incremental tests. During standard cycling tests, these parameters differ from those obtained during simulated ski mountaineering on a treadmill, indicating that the latter, which engage both upper- and lower body muscles, are more specific and reliable in this context (Schöffl et al., 2018).

Such sport-specific testing (e.g., a graded exercise test) has been carried out both on motorized treadmills (Schenk et al., 2011; Fornasiero et al., 2018; Lasshofer et al., 2021) and in the field (Duc et al., 2011; Cassirame et al., 2015). In most cases, assessment on a treadmill involves roller skiing (with boots and bindings), but, recently, Schöffl et al. (2018) suggested using skis with skins instead. Most incremental tests to exhaustion in the field involve skiing up a relatively unchanging incline (e.g., 8–24°) at *a* progressively faster speed (Duc et al., 2011; Cassirame et al., 2015; Schöffl et al., 2018; Lasshofer et al., 2021), although in some cases, both the speed and incline have been elevated (Mourot et al., 2014; Fornasiero et al., 2018).

Even though elite ski mountaineers spend more time at elevated altitud*es* than recreational athletes and are, therefore, probably better acclimatized, the reduction in *VO*_2__*max*_ of the elite skiers during an incremental test performed under hypoxic conditions is more pronounced. This confirms previous comparisons of the effects of hypoxia on elite and recreational endurance athletes (MacInnis et al., 2015) and underlines the importance of this environmental factor (Faiss et al., 2014). Moreover, the responses to normobaric and hypobaric hypoxia while skiing on a treadmill or exercising on a cycle ergometer differ and such differences must be taken into consideration when interpreting the results of exercise tests and/or monitoring athletic training (Treml et al., 2020).

## Equipment

**FIGURE 1:**
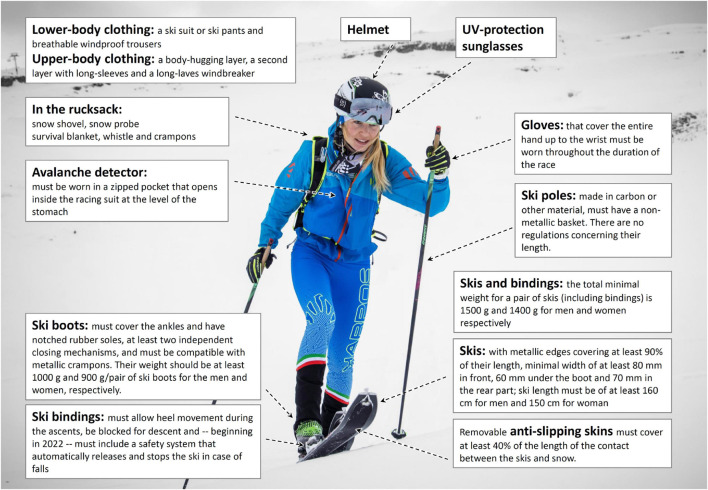
Major features of the equipment utilized during ski mountaineering competitions. Safety equipment, as well as clothing designed to protect against excessive cold, have must be worn. In addition, supplementary equipment and additional clothing may be required for specific events, depending on the characteristics of the course and/or weather conditions. All of this equipment must be borne throughout the race and may be inspected at any time (photo: Maurizio Torri).

The major features of ski mountaineering equipment, i.e., the skis, bindings, boots, and poles, are illustrated in Figure 1, and more specific features and up-to-date regulations are presented on the official ISMF website.^1^ To maximize safety, the athlete is required to utilize certain equipment throughout the entire competition (Figure 1).

Within the limits set by these regulations, companies attempt to develop equipment that balances reliability and lightness in an optimal fashion. Tosi et al. (2009) found that 1 kg of extra weight at the ankles of a ski mountaineer increases the energy cost by approximately 2–3%, which is negligible in the case of amateurs but may be decisive in elite competitions. To allow a long stride, the boots must allow extensive ankle flexion-extension during ascents, while, at the same time, being sufficiently stiff when locked during descents. A recent analysis of the race times during five consecutive Patrouille Des Glaciers competitions (2004–2014) attributed improvement in performance, in particular by members of the best teams, to the development of better equipment (Gasser, 2019b).

## Concluding Remarks and Future Perspectives

Considering the remarkable combination of extreme physiological and biomechanical/technical demands and exposure to challenging environments faced during ski mountaineering, it is surprising that so relatively little research has been performed on this sport in comparison to other winter endurance sports such as cross-country skiing.

Predominantly, physiological research on ski mountaineers has focused on determinants of performance, highlighting, among other aspects, the importance of high maximal oxygen uptake, as well as oxygen uptake at VT2. Since competitions frequently occur at altitudes above 1800 m and cold temperatures, investigations to optimize/improve training and overall performance should also, at least to some extent, include challenging environmental conditions. In light of the significant total elevation involved in a race, resulting in much of the total time being spent skiing uphill, the weight of the athlete and his/her equipment are especially important during ski mountaineering. Because of such differences, ski mountaineers may specialize even further in certain types of competitions, as is the case in several other endurance sports. Sex differences in ski mountaineering have been little studied and deserve considerably more attention.

The few investigations of various biomechanical aspects of ski mountaineering published to date often take only basic kinematic variables into consideration. Further insights into the biomechanics of this sport and appropriate development of equipment should be performed in collaboration with manufacturers. The recent introduction of smart devices designed specifically for monitoring ski mountaineering (Fasel et al., 2016; Gellaerts et al., 2018), in combination with data collected by GNSS, will allow a more systematic assessment of these biomechanical parameters.

In addition, the physiological demands associated with the varying effort required along a ski mountaineering race could also be evaluated in greater detail utilizing portable devices that provide additional information such as external load and pacing. Information of this sort would be of considerable value in assessing the wide variety of conditions (e.g., snow conditions, course characteristics, and hypoxia) to which a ski mountaineer is often exposed.

The recent inclusion of ski mountaineering as an Olympic event will enhance public awareness of and interest in this sport, allowing it to develop more rapidly than ever in the near future. The achievement of such progress clearly requires the combined efforts of the research community and active ski mountaineers.

## Ethics Statement

Written informed consent was obtained from the individual(s) for the publication of any potentially identifiable images or data included in this article.

## Author Contributions

LB, AS, BP, RM, MSa, H-CH, and MSu contributed to the conception and design of the manuscript. LB, AS, and MSu performed the literature search and wrote the first draft of the manuscript. LB, AS, BP, MSa, H-CH, and MSu wrote sections of the manuscript. LB, AS, BP, and MSa searched and organized the information for Table 1 and Figure 1. All authors contributed to the discussion section and manuscript revision, read, and approved the submitted version.

## Conflict of Interest

MSa was employed by Oberalp Group SPA, Italy. The remaining authors declare that the research was conducted in the absence of any commercial or financial relationships that could be construed as a potential conflict of interest. The handling editor declared a past co-authorship with one of the authors AS.

## Publisher’s Note

All claims expressed in this article are solely those of the authors and do not necessarily represent those of their affiliated organizations, or those of the publisher, the editors and the reviewers. Any product that may be evaluated in this article, or claim that may be made by its manufacturer, is not guaranteed or endorsed by the publisher.

## Footnotes

1
http://ismf-ski.org/
